# Etiological Study of Acute Conjunctivitis Caused by Human Adenovirus in Shanxi Province, China, between 2016 and 2019

**DOI:** 10.1128/spectrum.00159-23

**Published:** 2023-07-24

**Authors:** Jitao Wang, Xiaoling Ning, Yang Xu, Rui Wang, Xiaofang Guo, Jihong Xu, Jiane Guo, Qin Ma, Hong Li, Dandan Niu, Ying Liu, Naiying Mao, Zhen Zhu

**Affiliations:** a Department of Microbiology Test, Taiyuan Center for Disease Control and Prevention, Taiyuan, People’s Republic of China; b Comprehensive Inspection Section, Shanxi Eye Hospital, Taiyuan, People’s Republic of China; c Clinical Research Institute, The Affiliated Hospital of Southwest Medical University, Luzhou, People’s Republic of China; d NHC Key Laboratory of Medical Virology and Viral Diseases, National Institute for Viral Disease Control and Prevention, Chinese Center for Disease Control and Prevention, Beijing, People’s Republic of China; IrsiCaixa Institut de Recerca de la Sida

**Keywords:** acute conjunctivitis, etiological study, human adenovirus, human adenovirus type 85

## Abstract

Human adenovirus (HAdV) is the primary cause of acute conjunctivitis. To improve our understanding of the etiology of adenoviral conjunctivitis, ocular samples were collected from 160 conjunctivitis cases in the Shanxi province of northern China between 2016 and 2019. Through preliminary identification, virus isolation, and type identification, a total of 63 HAdV isolates were obtained from the samples. Three species and seven types (HAdV-3, HAdV-4, HAdV-8, HAdV-37, HAdV-53, HAdV-64, and HAdV-85) were detected, with HAdV-64, HAdV-3, and HAdV-8 being the predominant types in 2016, 2018, and 2019, respectively. Further phylogenetic analysis indicated the relative genomic stability of seven HAdV-type strains, except for 4 HAdV-3 strains in 2018 with a novel amino acid insertion site (Pro) between P19 and S20 in the penton base gene. It is worth noting that the genomes of two Shanxi HAdV-85 strains from 2016 were almost identical to those of previously reported HAdV-85 strains that circulated in Japan in 2014 to 2018. China was the second country to sample and isolate HAdV-85, suggesting that HAdV-85 might be underreported as an ocular pathogen. Data obtained in this study provide valuable information on the prevalence of acute conjunctivitis caused by HAdV.

**IMPORTANCE** HAdV types in cases of conjunctivitis in Shanxi province, China, in 2016 to 2019 showed evident diversity, with seven types (HAdV-3, HAdV-4, HAdV-8, HAdV-37, HAdV-53, HAdV-64, and HAdV-85) being identified, and relative genome stability of these viruses was observed. In addition, China was the second country to sample and isolate HAdV-85, which suggests that HAdV-85 might be underreported as an important pathogen associated with ocular infections. These results enhance the understanding of the etiology of adenoviral conjunctivitis and may aid in the development of prevention and control strategies for HAdV-related ocular infections in China.

## INTRODUCTION

Human adenovirus (HAdV), which belongs to the genus *Mastadenovirus* in the family *Adenoviridae*, is a nonenveloped, double-stranded DNA virus with a genome size of approximately 36 kb (1). Based on their biological properties, hemagglutination assay results, and genomic sequencing and analysis, HAdVs have been grouped into seven species (species A to G), containing at least 113 genotypes (2). Since genetic recombination of HAdV is the major driving force for molecular evolution (3), it is necessary to obtain the sequences of at least three capsid genes (penton base, hexon, and fiber genes) to accurately identify HAdV types. The standard nomenclature based on the three genes has also been established; for example, the type of HAdV-55 is P14H11F14 (penton base, HAdV-14; hexon, HAdV-11; fiber, HAdV-14) (http://hadvwg.gmu.edu).

HAdV is known to cause a wide variety of diseases, and the tissue tropism of specific virus species determines the clinical manifestations, including acute respiratory diseases (4, 5), gastroenteritis (6), conjunctivitis (7), and urethritis (8). Acute adenoviral conjunctivitis is a common infectious disease that occurs worldwide and accounts for approximately 65 to 90% of conjunctivitis cases (9). HAdV associated with ocular infections is typically self-limiting, while severe infections may lead to ocular lesions that can last from months to years (9). Viruses within species B (HAdV-3 and HAdV-7), D (HAdV-8, HAdV-37, HAdV-53, HAdV-54, HAdV-56, HAdV-64, and HAdV-85), and E (HAdV-4) have been reported to be associated with adenoviral conjunctivitis (9). Among them, HAdV-53 (P37H22F8), HAdV-54 (P54H54F8), HAdV-56 (P56H15F9), and HAdV-85 (P37H19F8) were identified as new types based on their genomic recombination events. HAdV-64 (P22H19F37) was formerly known as HAdV-19a and was renamed because of its recombinant pattern (10).

HAdV-associated conjunctivitis can lead to significant morbidity and increased medical expenditures. However, little is known about the distribution of HAdV types in conjunctivitis cases in China, due to lack of a systematic surveillance system. Therefore, this study collected ocular samples from conjunctivitis cases in Shanxi province in northern China between 2016 and 2019 and identified the HAdV types. This study aimed to improve the understanding of the etiology of adenoviral conjunctivitis and contribute to the development of prevention and control strategies for HAdV-related ocular infections in China.

## RESULTS

### HAdV identification and virus isolates.

A total of 160 patients from Shanxi province who were diagnosed with acute conjunctivitis between 2016 and 2019 were enrolled in this study. Among the 160 eye swab samples collected from the patients, 80 were identified as HAdV positive by real-time PCR assay, with a detection rate of 50%. After isolation of the virus, 63 HAdV isolates (21 each from 2016, 2017, and 2018) were obtained from the samples (see Table S2 in the supplemental material).

### Types of HAdV identified on the basis of three capsid genes.

**FIG 1:**
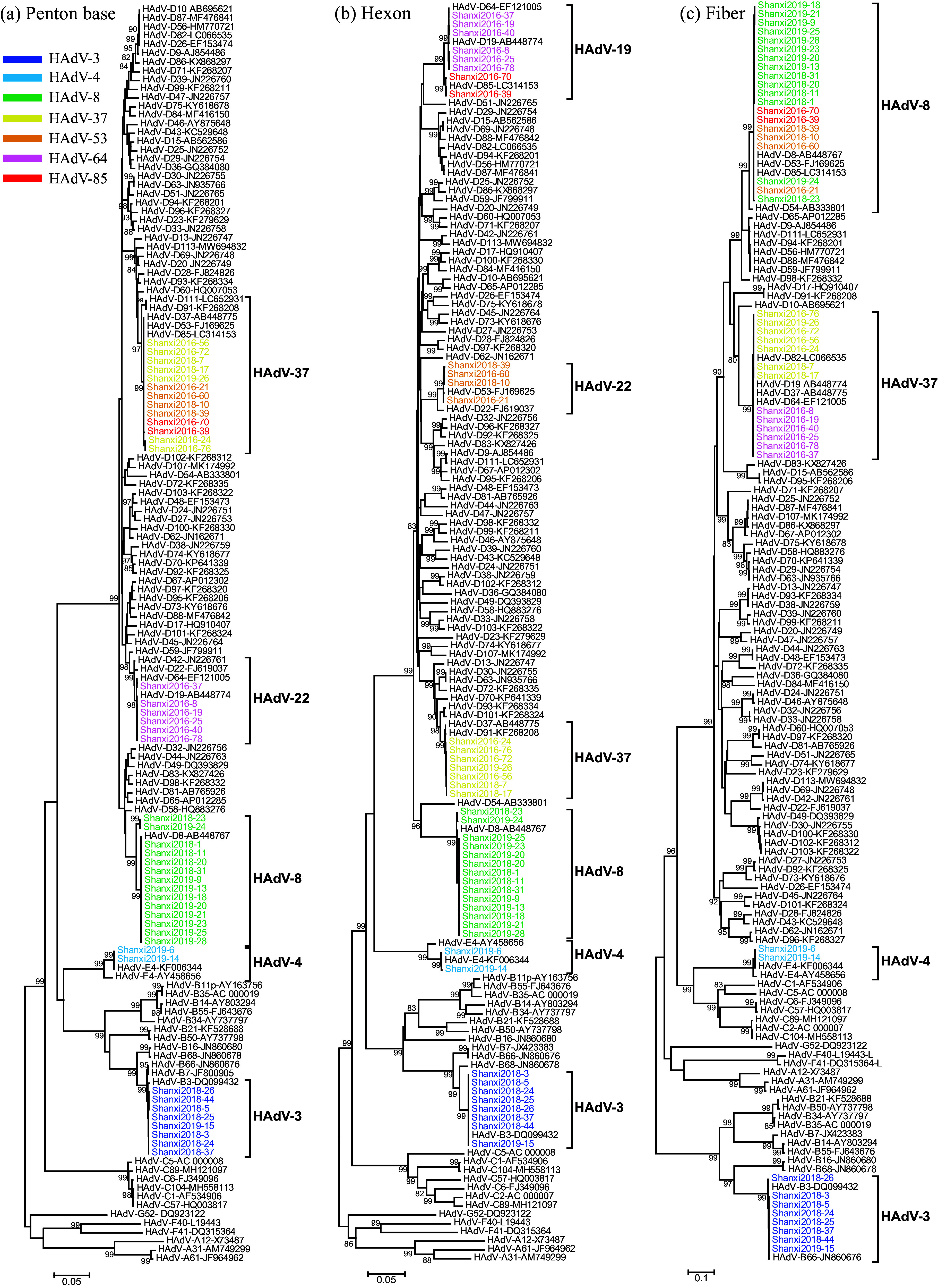
Type identification of Shanxi HAdV isolates based on penton base (a), hexon (b), and fiber (c) genes. To simplify the phylogenetic tree, virus strains in this study that were consistent across all three genes were removed, which resulted in the selection of 43 representative strains from 63 strains (HAdV-3, 8 strains; HAdV-8, 14 strains; HAdV-37, 7 strains; HAdV-53, 4 strains; HAdV-64, 6 strains; HAdV-85, 2 strains; HAdV-4, 2 strains) for construction of the phylogenetic tree. The representative strains of HAdV types in the trees were defined with HAdV type and GenBank accession number.

Three target genes, i.e., penton base, hexon, and fiber genes, were amplified from the virus isolates. Compared to 100 representative strains depicting 7 species (species A, 3 strains; species B, 11 strains; species C, 7 strains; species D, 74 strains; species E, 2 strains; species F, 2 strains; species G, 1 strain), the 63 Shanxi strains could be divided into 3 species and 7 types, including species B (HAdV-3 [P3H3F3], 10 strains in 2018 and 2019), species D (HAdV-8 [P8H8F8], 25 strains in 2018 and 2019; HAdV-37 [P37H37F37], 8 strains in 2016 to 2019; HAdV-53 [P37H22F8], 7 strains in 2016 and 2018; HAdV-64 [P22H19F37], 9 strains in 2016; HAdV-85 [P37H19F8], 2 strains in 2016), and species E (HAdV-4 [P4H4F4], 2 strains in 2019) (Fig. 1; also see Table S2). HAdV-8 had the highest detection rate (39.7%), followed by HAdV-3 (15.9%), HAdV-64 (14.3%), HAdV-37 (12.7%), HAdV-53 (11.1%), HAdV-85 (3.2%), and HAdV-4 (3.2%). However, different dominant types were detected each year. For example, HAdV-64 and HAdV-3 had the highest detection rates in 2016 (42.9%) and 2018 (42.9%), respectively, while HAdV-8 was dominant in 2019 (81.0%) (Table 1).

**TABLE 1:** HAdV types and age distribution of related cases in Shanxi province in 2016 to 2019

Species	Type	2016 (*n* = 21)		2018 (*n* = 21)		2019 (*n* = 21)		Total no. of isolates (%)
		No. of isolates (%)	Patient age (yr)	No. of isolates (%)	Patient age (yr)	No. of isolates (%)	Patient age (yr)	
B	HAdV-3	0 (0.0)		9 (42.9)	8–42	1 (4.8)	34	10 (15.9)
D	HAdV-8	0 (0.0)		8 (38.1)	32–56	17 (81.0)	20–76	25 (39.7)
	HAdV-37	5 (23.8)	27–49	2 (9.5)	26, 28	1 (4.8)	30	8 (12.7)
	HAdV-53	5 (23.8)	26–41	2 (9.5)	42, 46	0 (0.0)		7 (11.1)
	HAdV-64	9 (42.9)	14–52	0 (0.0)	26	0 (0.0)		9 (14.3)
	HAdV-85	2 (9.5)	42, 51	0 (0.0)		0 (0.0)		2 (3.2)
E	HAdV-4	0 (0.0)		0 (0.0)		2 (9.5)	35, 56	2 (3.2)

Further sequence identity analysis based on the penton base, hexon, and fiber genes showed that HAdV-3, HAdV-4, HAdV-37, HAdV-53, HAdV-64, and HAdV-85 strains isolated in Shanxi in 2016 to 2019 were more conserved and shared high levels of nucleotide identity (>99.7%, with a maximum of 6 nucleotide substitutions) and amino acid identity (>99.1%, with a maximum of 3 amino acid substitutions) with the corresponding types of HAdV. Interestingly, 4 HAdV-3 strains in 2018 had a novel insertion site (Pro) between P19 and S20 in the penton base gene, compared to the prototype strain (strain GB [GenBank accession number AY599834]). In comparison, HAdV-8 showed slightly greater sequence variation across the three genes, and the levels of similarity in the penton base, hexon, and fiber genes were 98.5 to 100% (0 to 22 nucleotide substitutions), 99.5 to 100% (0 to 14 nucleotide substitutions), and 99.4 to 100% (0 to 6 nucleotide substitutions), respectively.

### Clinical characteristics of HAdV type-related cases.

Sixty-three patients, with ages ranging between 8 and 76 years (median, 37 years), were infected with different HAdV types regardless of age differences (Table 1). Most cases presented with symptoms of acute infection. Eye redness and swelling (80 to 100%), along with eye discharge (50 to 100%), were the two main clinical symptoms with which patients presented. HAdV-3 infections also resulted in symptoms of respiratory infection, including sore throat (50%), fever (40%), and cough (30%). As the main pathogen of conjunctivitis, HAdV-8 infections caused more severe symptoms in patients, such as pseudomembrane formation (36%), conjunctival hyperemia (16%), and bloody eye secretion (12%). However, it is worth noting that 2 patients infected with HAdV-85 had mild symptoms, such as foreign body sensation (100%) and periauricular lymph node enlargement (100%), in addition to the common manifestations of acute conjunctivitis (eye redness, swelling, and eye discharge) (Table 2).

**TABLE 2:** Clinical symptoms of conjunctivitis cases caused by seven types of HAdV

Clinical symptom(s)	No. of cases (%)						
	HAdV-3 (*n* = 10)	HAdV-8 (*n* = 25)	HAdV-37 (*n* = 8)	HAdV-53 (*n* = 7)	HAdV-64 (*n* = 9)	HAdV-85 (*n* = 2)	HAdV-4 (*n* = 2)
Eye redness and swelling	10 (100)	20 (80)	8 (100)	7 (100)	9 (100)	2 (100)	2 (100)
Eye discharge	6 (60)	20 (80)	8 (100)	7 (100)	9 (100)	2 (100)	1 (50)
Foreign body sensation	1 (10)		1 (12.5)	1 (14.3)		2 (100)	
Periauricular lymph node enlargement	3 (30)	2 (8)				2 (100)	
Blurred vision	1 (10)						
Tears	1 (10)			2 (28.6)			
Pseudomembrane formation^*a*^		9 (36)					1 (50)
Conjunctival hyperemia^*a*^		4 (16)	1 (12.5)				
Bloody eye secretion^*a*^		3 (12)					
Corneal opacity^*a*^		1 (4)					
Chemosis^*a*^		1 (4)					
Fever	4 (40)	5 (20)	1 (12.5)				
Cough	3 (30)	1 (4)					
Sore throat	5 (50)	5 (20)	1 (12.5)				1 (50)
Enlarged tonsils		2 (8)					

a Severe symptom of HAdV infection.

### Phylogenetic analysis of seven HAdV types.

To further understand the genetic relationships among strains from Shanxi, other Chinese provinces, and other countries, the three target gene sequences of representative strains from 13 provinces of China from 2004 to 2020 and 13 countries from 1953 to 2020 were retrieved from the GenBank database (see Table S3).

**FIG 2:**
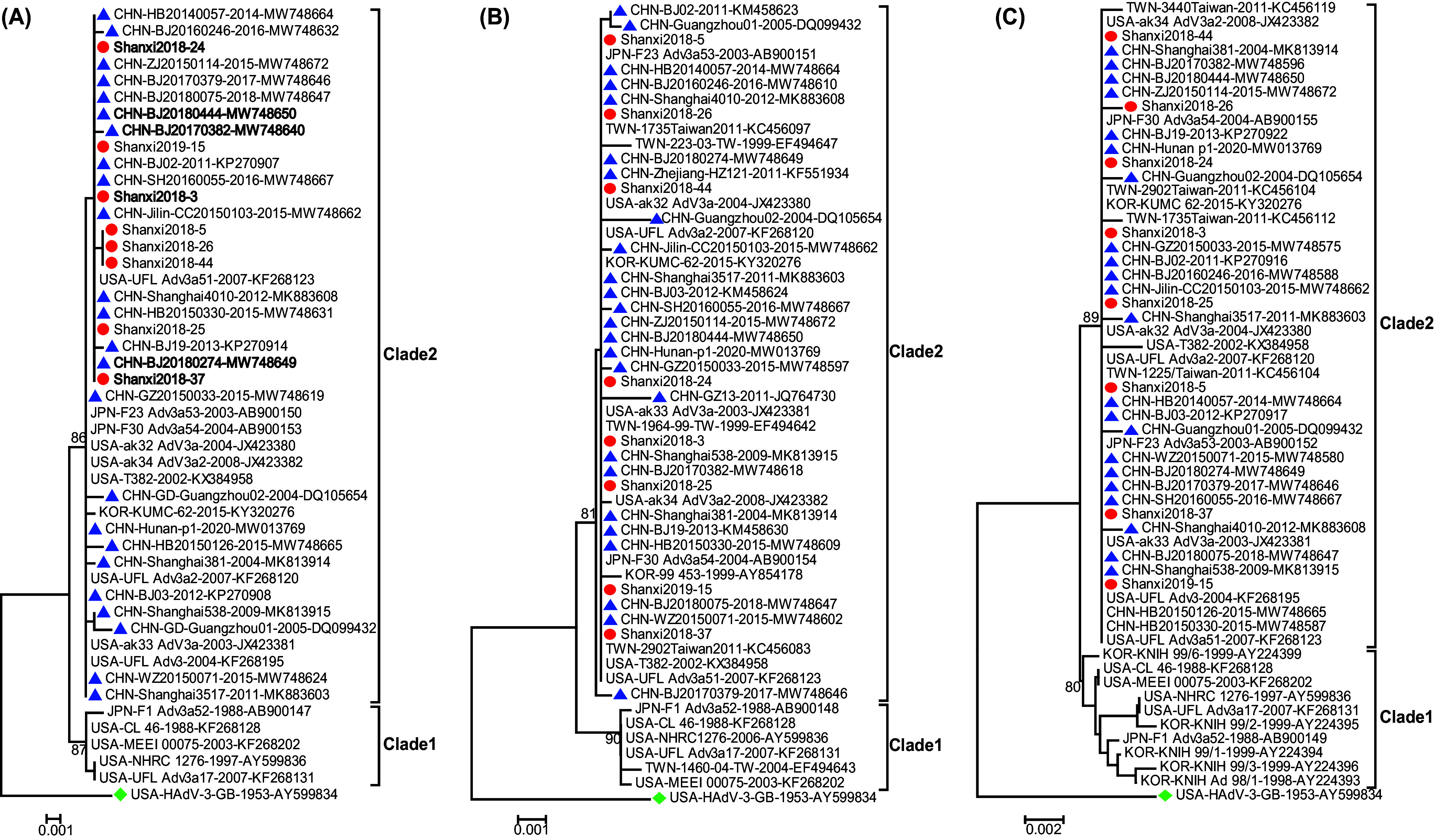
Phylogenetic analysis of species B strains (HAdV-3) based on penton base (A), hexon (B), and fiber (C) genes. Red circles represent the strains in this study, blue triangles represent strains circulating in other provinces of China, green diamonds represent the HAdV-3 prototype strain, and black bold type indicates HAdV-3 strains with an insertion site (Pro) between P19 and S20 in the penton base gene. HAdV-3 strains in this study that were consistent across all three genes were excluded; therefore, 8 representative strains were selected from 10 strains for construction of the phylogenetic tree. The representative strains from the GenBank database in the trees were defined with country of origin, strain name and GenBank accession number.

**FIG 3:**
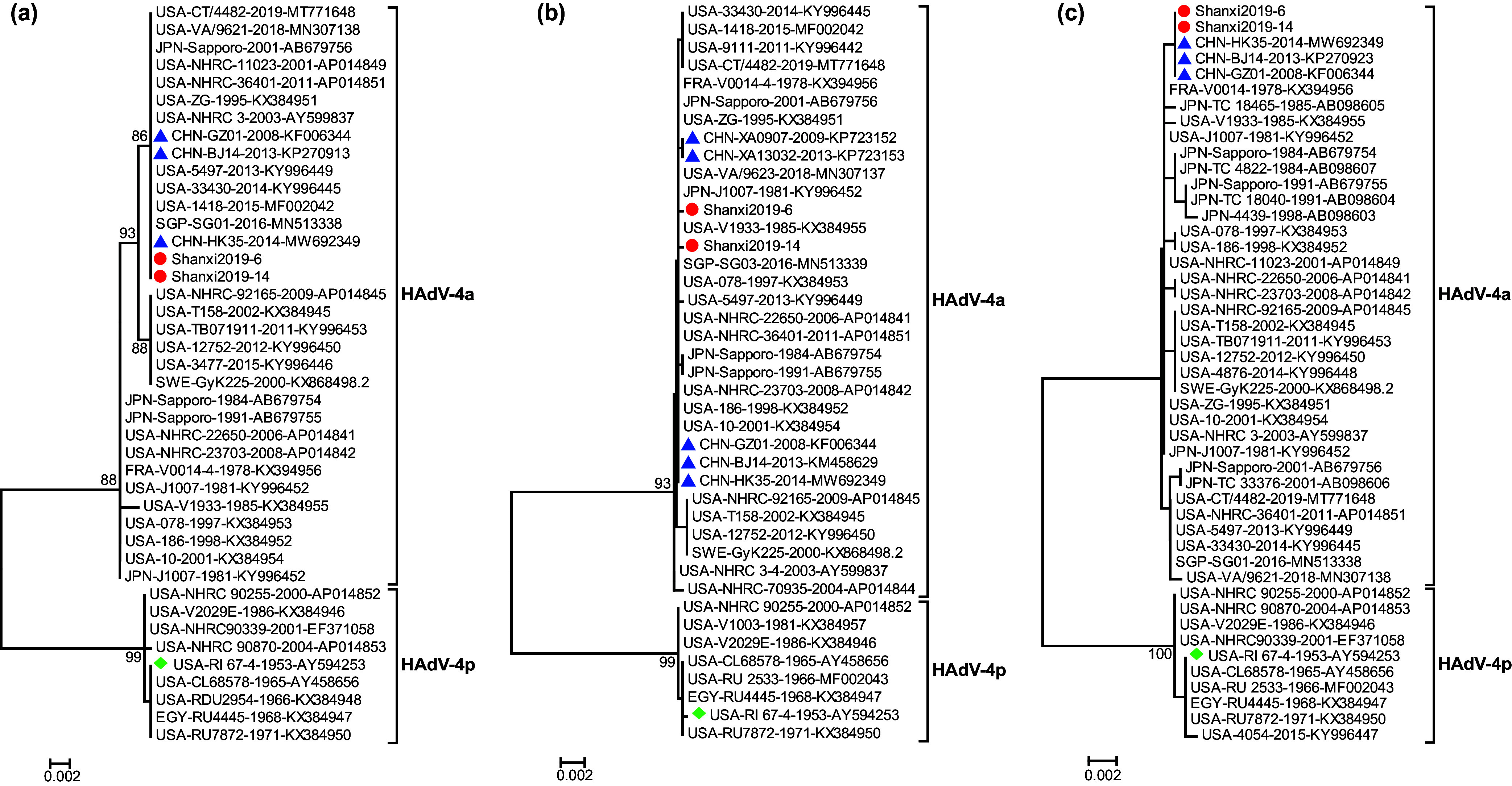
Phylogenetic analysis of species E strains (HAdV-4) based on penton base (a), hexon (b), and fiber (c) genes. Red circles represent the strains in this study, green diamonds represent the prototype stain of HAdV-4, and blue triangles represent strains circulating in other provinces of China. The representative strains from the GenBank database in the trees were defined with country of origin, strain name and GenBank accession number.

**FIG 4:**
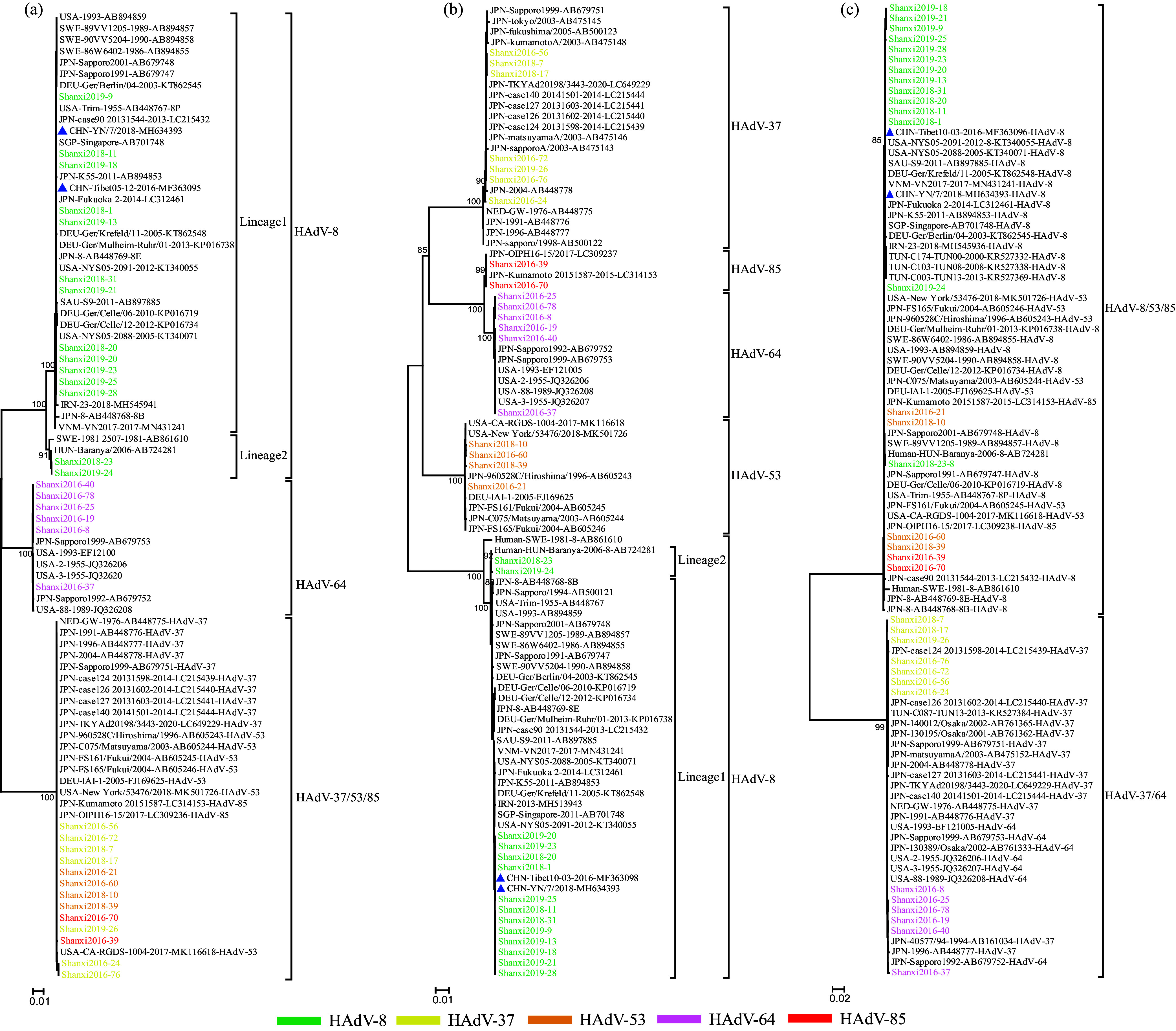
Phylogenetic analysis of species D strains (HAdV-8, HAdV-37, HAdV-53, HAdV-64, and HAdV-85) based on penton base (a), hexon (b), and fiber (c) genes. To simplify the phylogenetic tree, virus strains in this study that were consistent across all three genes were removed, which resulted in the selection of 33 representative strains from 51 strains (HAdV-8, 14 strains; HAdV-37, 7 strains; HAdV-53, 4 strains; HAdV-64, 6 strains; HAdV-85, 2 strains) for construction of the phylogenetic tree. Blue triangles represent strains circulating in other provinces of China. The representative strains from the GenBank database in the trees were defined with country of origin, strain name and GenBank accession number.

The results of phylogenetic analysis showed that the three capsid gene sequences of the strains in this study were similar to those of previous strains from patients of different ages and geographical regions. This indicated relative genome stability of the strains of seven HAdV types. (i) The HAdV-3 and HAdV-4 Shanxi strains belonging to clade 2 of HAdV-3 and HAdV-4a clustered with the dominant strains from other provinces of China and other countries in 2002 to 2020 (HAdV-3) and 1978 to 2019 (HAdV-4) (Fig. 2 and Fig. 3). The genetic distances between groups for the three genes for HAdV-3 and HAdV-4 were <0.002. In addition, the insertion site (Pro) in the penton base gene was observed in 3 HAdV-3 strains from Beijing city of China in 2017 and 2018 in this study, and the sequence identity between Shanxi and Beijing strains was 99.9 to 100%. (ii) For HAdV-8, Shanxi strains could be divided into two lineages (lineage 1 and lineage 2) according to the phylogenetic analysis of the penton base and hexon genes, with the support of a high bootstrap value (>80%). Within each lineage, Shanxi strains showed high sequence similarity to domestic and foreign strains between 1955 and 2018 (genetic distance, <0.002) (Fig. 4). The genetic distances between lineages were 0.014 (penton base gene) and 0.004 (hexon gene). In terms of fiber genes, all Shanxi strains showed high sequence identity with strains from other provinces of China and other countries in 1955 to 2018, and the genetic distance between groups was <0.002. (iii) For HAdV-37 and 3 recombinant strains (HAdV-53, HAdV-64, and HAdV-85), Shanxi strains also showed high sequence similarity among the three genes with respect to strains of related types in other countries in 1976 to 2020 (HAdV-37), 1989 to 2018 (HAdV-53), 1955 to 2002 (HAdV-64), and 2015 and 2017 (HAdV-85) (genetic distance, <0.001) (Fig. 4).

### Whole-genome sequencing and analysis of 2 HAdV-85 strains.

**FIG 5:**
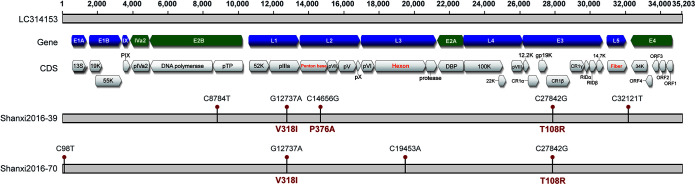
Comparative whole-genome-sequence-based analysis of two Shanxi HAdV-85 strains and the HAdV-85 prototype strain from Japan (GenBank accession number LC314153). CDS, coding sequence; ORF, open reading frame; DBP, DNA-binding protein; RID, receptor internalization and degradation protein.

To investigate the genomic characteristics of 2 HAdV-85 strains that were isolated from Shanxi province in 2016, their whole genomes were sequenced and analyzed. Consistent with the prototype strain of HAdV-85 reported from Japan (strain Kumamoto_20151587_JPN [GenBank accession number LC314153]) (11), the genome sizes of both Shanxi strains (Shanxi2016-39 and Shanxi2016-70) were 35,203 bp, and a total of 34 putative coding regions were identified for the Shanxi strains (Fig. 5).

Comparative analysis showed high similarity (>99.9%) of the genome sequences of the Shanxi strains and the HAdV-85 prototype strain, with only 4 or 5 nucleotide substitutions and 2 or 3 amino acid substitutions. Two Shanxi strains shared 2 amino acid mutations, i.e., V318I in the pIIIa region of the L1 gene and T108R in the CR1-β region of the E3 gene (Fig. 5).

## DISCUSSION

HAdV is the main cause of infectious conjunctivitis, while endemic HAdV associated with ocular infections has rarely been reported due to insufficient surveillance. In this study, 160 patients from Shanxi province who were diagnosed with acute conjunctivitis between 2016 and 2019 were enrolled. The HAdV detection rate was 50%, which was comparable to reports from Brazil (60%) (12), Turkey (44%) (13), and Vietnam (55.83%) (14). Furthermore, HAdV types associated with ocular infections, including species B (HAdV-3), species D (HAdV-8, HAdV-37, HAdV-53, HAdV-64, and HAdV-85), and species E (HAdV-4), were identified in this study, with HAdV-64, HAdV-3, and HAdV-8 being predominant in 2016, 2018, and 2019, respectively. High levels of divergence and polymorphism in ocular HAdV types were reported by other studies, whereas the dominant HAdV types varied among regions or countries. This might be due to the differences in time periods and geographical areas investigated (12–16).

It is worth noting that no trend was observed in terms of the age distribution of patients infected with different types of HAdV in this study. Most of the patients presented with common clinical manifestations, such as ocular redness, swelling, and eye discharge, which made distinguishing this entity from other diseases, such as bacterial conjunctivitis and allergic conjunctivitis, an arduous task in clinical practice. Although there is no specific and effective treatment for adenoviral conjunctivitis and its complications, timely identification of the virus could reduce unnecessary use of antibiotics, and targeted antiviral therapy could shorten the course of infection and further reduce the risk of complications. In addition, since all age groups are susceptible to ocular infections and extracellular HAdV is highly stable, good hygiene practices, such as frequent hand washing and disinfection of instruments, are essential to prevent the spread of viral infection (9).

The mutation rates of several RNA viruses, such as coronavirus and influenza virus, are disastrously high. High-frequency mutations could provide opportunities for the viruses to escape the host immune response (17, 18). However, HAdV, with a DNA genome, is relatively stable, and very similar sequences of three capsid genes were found decades apart for the HAdV types in this study. Limited mutations and genetic drift provide an important basis for the safe use of adenoviral vectors and the development of efficient HAdV vaccines. Interestingly, HAdV-3 strains with a novel amino acid insertion site (Pro) between P19 and S20 in the penton base gene observed in 2018 in this study were also found in acute respiratory infection cases from Beijing, China, during the same period, indicating that HAdV-3 strains with the novel insertion site had spread in China as the pathogen for both respiratory infections and conjunctivitis. Penton base can bind to the integrin entry receptor and exploit integrin-mediated signaling to enter the cell via endocytosis (19). Whether this novel insertion has an effect on that function requires further investigation.

Apart from genome stability across time and space, homologous recombination has been confirmed as the major pathway in molecular evolution of HAdV (3). “Nonpathogenic” or “low-pathogenic” viruses evolve into highly contagious variants through recombination. In addition to HAdV-53 and HAdV-64, which were identified in the past decade, HAdV-85, designated based on the combination of HAdV-37 (penton base), HAdV-19 (hexon), and HAdV-8 (fiber), is a new recombinant virus that was identified in recent years (11). HAdV-85 was first reported as the causative agent of epidemic keratoconjunctivitis (EKC) in Kumamoto city in Japan between 2014 and 2015 (11), and then it was detected again from suspected EKC cases in Fukushima prefecture in Japan between 2017 and 2018 (20). Most cases of HAdV-85 infection present with severe adenoviral conjunctivitis (11, 20). The genomes of two Shanxi HAdV-85 strains in 2016 were almost identical to those of previously reported HAdV-85 strains that circulated in Japan between 2014 and 2018. To the best of our knowledge, China was the second country to sample and isolate HAdV-85, which suggests that HAdV-85 might be underreported as an important ocular pathogen, particularly if only one of the three capsid genes was used for typing. Notably, in addition to ocular infection, HAdV-85 can be isolated from urethritis cases (21). The tissue tropism of HAdV-85 might present diversity due to genetic recombination, thereby highlighting the potential risk to public health. Accordingly, the epidemiology of HAdV-85 should be carefully monitored, both nationally and globally.

This study had few inherent limitations. For instance, it is difficult to distinguish cases of EKC, pharyngeal conjunctival fever, and acute hemorrhagic conjunctivitis due to their similar clinical presentations. In addition, because adenovirus-related conjunctivitis is not a notifiable reported infectious disease in China, continuous surveillance of HAdV-related conjunctivitis cases was not carried out in this study. Therefore, the total number of conjunctivitis cases among outpatients could not be determined, and the disease burden could not be understood. In addition, continuous sampling of conjunctivitis cases was not performed; therefore, conjunctivitis-associated HAdV types could not be fully understood, and the seasonality of HAdV could not be determined in this study.

In conclusion, seven adenovirus types were identified in conjunctivitis cases in Shanxi province from 2016 to 2019, and phylogenetic analysis indicated genetic conservation and stability of these HAdV types. However, the data obtained in this study were relatively limited and could not reflect the overall situation in China. Based on these findings, it is necessary to carry out systematic surveillance of conjunctivitis-associated HAdV strains across chronological periods and geographical areas. This would help delineate the epidemic and evolutionary patterns of adenoviral conjunctivitis and related viruses and provide a scientific basis for the prevention and control of HAdV-associated conjunctivitis in China.

## MATERIALS AND METHODS

### Case definition and ethics statement.

Acute conjunctivitis was defined as any inflammation of the conjunctiva characterized by redness of the eye, and its symptoms may include pain, itching, and foreign body sensation, accompanied by tears or increased secretion (15). All participants who met the case definition were required to provide written informed consent for the use of their clinical information and samples for the study.

This study was approved by the second session of the Ethics Review Committee of the National Institute for Viral Disease Control and Prevention (IVDC) at the Chinese Center for Disease Control and Prevention (CDC). All methods in this study were performed according to the relevant guidelines and regulations.

### Sample collection, identification, and virus isolation.

A total of 160 eye swabs were collected from outpatients who had been clinically diagnosed with acute conjunctivitis in January to September 2016 (79 samples), November to December 2018 (45 samples), and April to September 2019 (36 samples) at Shanxi Eye Hospital, located in Taiyuan city, Shanxi province, China. Samples were transported to IVDC, under cold chain conditions, for further pathogenic identification. Viral DNA was extracted from the clinical samples by using a nucleic acid extraction kit and a Generotex 96 automatic nucleic acid extractor (Tianlong, Shaanxi, China) according to the manufacturer's instructions. After the viral DNA was extracted, HAdV identification was performed using a previously reported real-time PCR method (22).

All HAdV-positive clinical samples were inoculated in human laryngeal epidermoid carcinoma (HEp-2) cells maintained in Dulbecco’s modified Eagle medium (DMEM) supplemented with 2% fetal bovine serum. The cultures were observed for 5 to 7 days for HAdV cytopathic effect (CPE) and harvested when approximately 75 to 80% of the cells exhibited specific CPE. Three consecutive passages were required for samples without CPE.

### Amplification and sequencing of three target genes.

Nucleotide sequences of the penton base, hexon, and fiber genes of virus isolates were determined using in-house primer pairs specific for each HAdV type (see Table S1 in the supplemental material) and Platinum PCR SuperMix (Invitrogen, Carlsbad, CA, USA). Positive PCR products were sequenced using the traditional Sanger method after purification, and ≥2-fold coverage in both directions was used to obtain high-quality sequences. Raw sequence data were assembled and edited using Sequencher version 5.0 (Gene Codes Corp., Ann Arbor, MI, USA).

### Whole-genome sequencing and annotation.

Twenty overlapping PCR fragments of HAdV-85, covering the full-length genome, were amplified using in-house primer pairs (see Table S1) and Platinum PCR SuperMix (Invitrogen). Furthermore, amplified DNA was used as a template for sequencing using the Sanger method and an ABI Prism 3100 Genetic Analyzer (Life Technologies, Japan). Questionable sites were resequenced to ensure accuracy.

### Bioinformatic analysis.

Multiple sequence alignments were performed using MAFFT version 7.490 (23) (https://mafft.cbrc.jp/alignment/software). Phylogenetic analysis of aligned sequences was performed using MEGA version 11.0.11 (24) (https://www.megasoftware.net). Sequence similarity and genetic distances between or within groups were calculated using BioEdit version 7.0.9.0 and MEGA version 11.0.11, respectively. The whole-genome sequence was displayed and annotated using Geneious Prime version 2022.0.2.

### Data availability.

The nucleotide sequences of three capsid genes representing six types (HAdV-3, HAdV-4, HAdV-8, HAdV-37, HAdV-53, and HAdV-64) from 61 strains isolated from acute conjunctivitis cases in Shanxi province of China in 2016 to 2019 were deposited in the GenBank database with the accession numbers OQ128143 to OQ128325. The whole-genome sequences of two HAdV-85 strains in this study were submitted to GenBank with the accession numbers OQ128326 and OQ128327.
