# Supplementary material for: Etiological Study of Acute Conjunctivitis Caused by Human Adenovirus in Shanxi Province, China, between 2016 and 2019
Source: Microbiol Spectr. 2023 Jul 24;11(4):e00159-23. doi: 10.1128/spectrum.00159-23 (PMC10434163; doi:10.1128/spectrum.00159-23)
Supplement: Supplemental file 1 — Table S1 to S4. Download spectrum.00159-23-s0001.docx, DOCX file, 0.06 MB [file spectrum.00159-23-s0001.docx]

Supplementary Table1. Primers used for amplifying gene sequences of HAdV associated with acute conjunctivitis ^a^

| HAdV type | Primer | Sequences (5′-3′ orientation) | Position | Reference strain/  Accession number |
| --- | --- | --- | --- | --- |
| HAdV-37^b^ | HAdV37-Penton-F | ATAGCAGCGTGTTGGACTTG | 13,392–13,411 | GW/AB448775 |
|  | HAdV37-Penton-R | GCTGGGAGCGTTTCTTGG | 15,167–15,184 |  |
|  | HAdV37-Hexon-1F | AGTGAAAAGTCTGAAGCGCC | 17,702–17,721 |  |
|  | HAdV37-Hexon-1R | ACTTTTGGGGCACTTGGATG | 19,443–19,462 |  |
|  | HAdV37-Hexon-2F | ACTCCTACAAGTACACGCCG | 19,214–19,233 |  |
|  | HAdV37-Hexon-2R | GGTCGAACATGTAGCAGGTG | 20,825–20,844 |  |
|  | HAdV37-Fiber-F | CTCTGGTGTAGTTGYTCAGC | 30,897–30,916 |  |
|  | HAdV37-Fiber-R | CAAGTCCAATTCCCGCACTA | 32,289–32,308 |  |
| HAdV-64^b^ | HAdV64-Penton-F | ATAGCAGCGTGTTGGACTTG | 13,398-13,417 | human/USA/C/1993/64[P22H19F37]/ EF121005 |
|  | HAdV64-Penton-R | CCACCACGCTGTCRATGAC | 15,340-15,358 |  |
|  | HAdV64-Hexon-1F | AATGAAACCCGCCACCCTT | 17,522-17,540 |  |
|  | HAdV64-Hexon-1R | TCCCCGTCTTCACCATCTTG | 19,072-19,091 |  |
|  | HAdV64-Hexon-2F | AGGCCTCTCAGTTGAATGCT | 18,830-18,849 |  |
|  | HAdV64-Hexon-2R | GGTCGAACATGTAGCAGGTG | 20,846-20,865 |  |
|  | HAdV64-Fiber-F | CTCTGGTGTAGTTGYTCAGC | 30,913-30,932 |  |
|  | HAdV64-Fiber-R | CGTGCTGAGAGAACCGTGTA | 32,256-32,275 |  |
| HAdV-85^c^ | HAdV85-1F | CATCATCAATAATATACCCC | 1-20 | Kumamoto_20151587_JPN/ LC314153 |
|  | HAdV85-1R | GCTCCTCTCCCTCTTCACTC | 2,184-2,203 |  |
|  | HAdV85-2F | CAGGGGCTACATTCTGGACT | 1,907-1,926 |  |
|  | HAdV85-2R | TCCACCGCACTCTCAATGAT | 3,948-3,967 |  |
|  | HAdV85-3F | CATCATCGCCGAGGAGAAAC | 3,736-3,755 |  |
|  | HAdV85-3R | CGGAAGAGAGTGAGGACGAG | 5,849-5,868 |  |
|  | HAdV85-4F | TTACCTCGCGTCTCCATGAG | 5,511-5,530 |  |
|  | HAdV85-4R | TCACCTTCTACTGCGTCACA | 7,586-7,605 |  |
|  | HAdV85-5F | TGCCCGACGATGTAGAGTTC | 7,356-7,375 |  |
|  | HAdV85-5R | GCCCTACACCGCAATTGATA | 9,361-9,380 |  |
|  | HAdV85-6F | CCTCTTCTTCCATGACGACCT | 9,108-9,128 |  |
|  | HAdV85-6R | TTTTGGAAGTTGCGCTCCTG | 11,059-11,078 |  |
|  | HAdV85-7F | CGAGCAGACGGGGATCAG | 10,980-10,997 |  |
|  | HAdV85-7R | TTTTGCAAGGTCGGTGAGAA | 13,034-13,053 |  |
|  | HAdV85-8F | CGCAACATGGAACCTAGCAT | 12,821-12,840 |  |
|  | HAdV85-8R | GGATGAGCTGGGAGTAGACG | 14,814-14,833 |  |
|  | HAdV85-9F | GAATGATGCCGTCCCAAGAG | 14,439-14,458 |  |
|  | HAdV85-9R | TGATGGAAGGATGCAACACG | 16,520-16,539 |  |
|  | HAdV85-10F | GAAAATGGATGTGGAGCCCG | 16,265-16,284 |  |
|  | HAdV85-10R | TTGTTCCTCCCATAGCAGCC | 18,276-18,295 |  |
|  | HAdV85-11F | GGCCAGCACTTACTTTGACA | 18,093-18,112 |  |
|  | HAdV85-11R | AAGTTGCTGGAGAAGGGGAT | 20,419-20,438 |  |
|  | HAdV85-12F | CAGCACAACAACTCTGGCTT | 20,266-20,285 |  |
|  | HAdV85-12R | GCAGACCGAGATCTTCCAGA | 22,292-22,311 |  |
|  | HAdV85-13F | CCATGCAGACACTTGAGCTG | 22,081-22,100 |  |
|  | HAdV85-13R | GGTTCTCGTGTAGGATCCCC | 23,901-23,920 |  |
|  | HAdV85-14F | ACCCTGCACTACACTTTCAGA | 23,815-23,835 |  |
|  | HAdV85-14R | CGCGGAAGTAGTACGGTAGT | 25,788-25,807 |  |
|  | HAdV85-15F | CACCCCTTACATGTGGAGCT | 25,547-25,566 |  |
|  | HAdV85-15R | AGCCCACCATGTAGCATTGA | 27,644-27,663 |  |
|  | HAdV85-16F | TTTGTGATCATGGCCTGCTT | 27,444-27,463 |  |
|  | HAdV85-16R | TCATTGGCACGATCGTTTCA | 29,453-29,472 |  |
|  | HAdV85-17F | CCGCCAACTACTAAAGCACC | 29,232-29,251 |  |
|  | HAdV85-17R | CGACTGCTGGGTGATGACTA | 30,620-30,639 |  |
|  | HAdV85-18F | CCATCAACATCCACCAGTGC | 30,464-30,483 |  |
|  | HAdV85-18R | ACCAGACTGTGACCTTGGAC | 32,354-32,373 |  |
|  | HAdV85-19F | CGGTGATTGAGATGAAGCCG | 32,302-32,321 |  |
|  | HAdV85-19R | TACTCCCTTACGCTTGCCAG | 34,057-34,076 |  |
|  | HAdV85-20F | CACTTAAGTCCTGGGCCACT | 34,001-34,020 |  |
|  | HAdV85-20R | GAAAAACACCTAGGGCGTGT | 34,841-34,860 |  |

^a^ Since the primers for amplifying the three genes of human HAdV-3, HAdV-4, HAdV-8, and HAdV-53 had been published, they were not listed in the table.

^b^ Primers for amplifying three capsid genes were listed.

^c^ Primers for amplify whole genome sequence of HAdV-85 were listed.

Supplementary Table 2. Seventy-five cases associated with acute conjunctivitis in Shanxi province of China during 2016-2019

| No. | Sample ID | Gender | Age | Main clinical symptoms | | | | | | | | | | | | | Date of onset | HAdV type identification | |
| --- | --- | --- | --- | --- | --- | --- | --- | --- | --- | --- | --- | --- | --- | --- | --- | --- | --- | --- | --- |
|  |  |  |  | Eye redness and swelling | Secretion increased | Bloody secretion | Foreign body sensation | Pseudome-mbranous | Periauricular lymph node enlargement | Conjunctival hyperemia | Tears | Fever | Cough | Sore throat | Enlarged tonsils | others |  | Genotyping based on three genes | Genbank No. |
| 1 | Shanxi2016-8 | Female | 30 | √ | √ |  |  |  |  |  |  |  |  |  |  |  | 2016.01.11 | HAdV-64  [P22H19F37] |  |
| 2 | Shanxi2016-19 | Female | 14 | √ | √ |  |  |  |  |  |  |  |  |  |  |  | 2016.05.10 | HAdV-64 [P22H19F37] |  |
| 3 | Shanxi2016-21 | Male | 41 | √ | √ |  |  |  |  |  |  |  |  |  |  |  | 2016.05.13 | HAdV-53 [P37H22F8] |  |
| 4 | Shanxi2016-23 | Female | 39 | √ | √ |  |  |  |  |  |  |  |  |  |  |  | 2016.05.30 | HAdV-53 [P37H22F8] |  |
| 5 | Shanxi2016-24 | Female | 27 | √ | √ |  |  |  |  |  |  |  |  |  |  |  | 2016.05.30 | HAdV-37 [P37H37F37] |  |
| 6 | Shanxi2016-25 | Female | 27 | √ | √ |  |  |  |  |  |  |  |  |  |  |  | 2016.05.31 | HAdV-64 [P22H19F37] |  |
| 7 | Shanxi2016-29 | Male | 44 | √ | √ |  |  |  |  |  |  |  |  |  |  |  | 2016.06.03 | HAdV-64 [P22H19F37] |  |
| 8 | Shanxi2016-37 | Female | 52 | √ | √ |  |  |  |  |  |  |  |  |  |  |  | 2016.07.26 | HAdV-64 [P22H19F37] |  |
| 9 | Shanxi2016-38 | Female | 40 | √ | √ |  |  |  |  |  |  |  |  |  |  |  | 2016.07.28 | HAdV-53 [P37H22F8] |  |
| 10 | Shanxi2016-39 | Female | 51 | √ | √ |  | √ |  |  |  |  |  |  |  |  |  | 2016.07.29 | HAdV-85 [P37H19F8] |  |
| 11 | Shanxi2016-40 | Male | 35 | √ | √ |  |  |  |  |  |  |  |  |  |  |  | 2016.07.30 | HAdV-64 [P22H19F37] |  |
| 12 | Shanxi2016-51 | Male | 39 | √ | √ |  |  |  |  |  |  |  |  |  |  |  | 2016.08.30 | HAdV-53 [P37H22F8] |  |
| 13 | Shanxi2016-56 | Male | 34 | √ | √ |  |  |  |  |  |  |  |  |  |  |  | 2016.08.30 | HAdV-37 [P37H37F37] |  |
| 14 | Shanxi2016-60 | Male | 26 | √ | √ |  |  |  |  |  |  |  |  |  |  |  | 2016.08.30 | HAdV-53 [P37H22F8] |  |
| 15 | Shanxi2016-61 | Male | 29 | √ | √ |  |  |  |  |  |  |  |  |  |  |  | 2016.09.13 | HAdV-64 [P22H19F37] |  |
| 16 | Shanxi2016-63 | Male | 35 | √ | √ |  |  |  |  |  |  |  |  |  |  |  | 2016.09.13 | HAdV-37 [P37H37F37] |  |
| 17 | Shanxi2016-70 | Male | 42 | √ | √ |  | √ |  |  |  |  |  |  |  |  |  | 2016.09.22 | HAdV-85 [P37H19F8] |  |
| 18 | Shanxi2016-72 | Male | 40 | √ | √ |  |  |  |  |  |  |  |  |  |  |  | 2016.09.22 | HAdV-37 [P37H37F37] |  |
| 19 | Shanxi2016-75 | Male | 30 | √ | √ |  |  |  |  |  |  |  |  |  |  |  | 2016.09.22 | HAdV-64 [P22H19F37] |  |
| 20 | Shanxi2016-76 | Female | 49 | √ | √ |  |  |  |  |  |  |  |  |  |  |  | 2016.09.22 | HAdV-37 [P37H37F37] |  |
| 21 | Shanxi2016-78 | Male | 29 | √ | √ |  |  |  |  |  |  |  |  |  |  |  | 2016.07.20 | HAdV-64 [P22H19F37] |  |
| 22 | Shanxi2018-1 | Female | 56 |  |  |  |  | √ |  |  |  |  |  |  |  |  | 2018.12.03 | HAdV-8 [P8H8F8] |  |
| 23 | Shanxi2018-2 | Female | 34 |  |  | √ |  | √ |  |  |  |  |  |  |  |  | 2018.11.22 | HAdV-8 [P8H8F8] |  |
| 24 | Shanxi2018-3 | Female | 34 | √ | √ |  |  |  |  |  |  |  |  |  |  |  | 2018.11.30 | HAdV-3 [P3H3F3] |  |
| 25 | Shanxi2018-5 | Female | 28 | √ | √ |  | √ |  |  |  |  |  |  |  |  | Blurred vision | 2018.12.02 | HAdV-3 [P3H3F3] |  |
| 26 | Shanxi2018-7 | Male | 28 | √ | √ |  |  |  |  | √ |  |  |  |  |  |  | 2018.11.29 | HAdV-37 [P37H37F37] |  |
| 27 | Shanxi2018-10 | Male | 46 | √ | √ | √ |  |  |  |  | √ |  |  |  |  |  | 2018.12.07 | HAdV-53 [P37H22F8] |  |
| 28 | Shanxi2018-11 | Male | 32 | √ |  |  |  |  |  |  |  |  |  |  |  |  | 2018.12.10 | HAdV-8 [P8H8F8] |  |
| 29 | Shanxi2018-15 | Male | 31 | √ |  |  |  |  |  |  |  |  |  |  |  |  | 2018.12.10 | HAdV-3 [P3H3F3] |  |
| 30 | Shanxi2018-17 | Male | 26 | √ | √ |  | √ |  |  |  |  |  |  |  |  |  | 2018.12.10 | HAdV-37 [P37H37F37] |  |
| 31 | Shanxi2018-20 | Female | 38 | √ | √ |  |  | √ |  |  |  |  |  |  |  |  | 2018.12.07 | HAdV-8 [P8H8F8] |  |
| 32 | Shanxi2018-23 | Male | 32 |  | √ |  |  |  |  |  |  |  |  |  |  |  | 2018.12.07 | HAdV-8 [P8H8F8] |  |
| 33 | Shanxi2018-24 | Male | 31 | √ | √ |  |  |  |  |  |  | √ |  | √ |  |  | 2018.12.13 | HAdV-3 [P3H3F3] |  |
| 34 | Shanxi2018-25 | Male | 30 | √ |  |  |  |  | √ |  |  |  |  | √ |  |  | 2018.12.14 | HAdV-3 [P3H3F3] |  |
| 35 | Shanxi2018-26 | Male | 8 | √ |  |  |  |  |  |  | √ | √ | √ |  |  |  | 2018.12.15 | HAdV-3 [P3H3F3] |  |
| 36 | Shanxi2018-31 | Female | 33 | √ | √ |  |  |  |  |  |  |  |  |  |  |  | 2018.12.08 | HAdV-8 [P8H8F8] |  |
| 37 | Shanxi2018-34 | Male | 43 | √ | √ |  |  |  |  |  |  |  |  |  |  | Corneal opacity | 2018.12.08 | HAdV-8 [P8H8F8] |  |
| 38 | Shanxi2018-37 | Male | 29 | √ | √ |  |  |  |  |  |  |  | √ | √ |  |  | 2018.12.21 | HAdV-3 [P3H3F3] |  |
| 39 | Shanxi2018-38 | Male | 42 | √ | √ |  |  |  | √ |  |  | √ |  |  |  |  | 2018.12.17 | HAdV-3 [P3H3F3] |  |
| 40 | Shanxi2018-39 | Female | 33 | √ | √ |  |  |  |  |  | √ |  |  |  |  |  | 2018.12.19 | HAdV-53 [P37H22F8] |  |
| 41 | Shanxi2018-42 | Female | 36 | √ | √ |  |  | √ |  | √ |  |  |  |  | √ |  | 2018.12.25 | HAdV-8 [P8H8F8] |  |
| 42 | Shanxi2018-44 | Female | 31 | √ | √ |  |  |  |  |  |  |  | √ | √ |  |  | 2018.12.26 | HAdV-3 [P3H3F3] |  |
| 43 | Shanxi2019-6 | Female | 35 | √ | √ |  |  | √ |  |  |  |  |  |  |  |  | 2019.04.18 | HAdV-4 [P4H4F4] |  |
| 44 | Shanxi2019-7 | Female | 26 | √ | √ |  |  |  |  |  |  |  |  |  |  |  | 2019.04.18 | HAdV-8 [P8H8F8] |  |
| 45 | Shanxi2019-8 | Male | 49 | √ | √ |  |  |  |  |  |  |  | √ | √ |  |  | 2019.04.22 | HAdV-8 [P8H8F8] |  |
| 46 | Shanxi2019-9 | Female | 35 | √ | √ |  |  |  |  |  |  |  |  |  |  |  | 2019.04.22 | HAdV-8 [P8H8F8] |  |
| 47 | Shanxi2019-11 | Female | 50 | √ | √ |  |  |  |  | √ |  |  |  |  |  | Chemosis | 2019.04.22 | HAdV-8 [P8H8F8] |  |
| 48 | Shanxi2019-12 | Female | 21 | √ | √ |  |  |  |  | √ |  |  |  |  |  |  | 2019.04.22 | HAdV-8 [P8H8F8] |  |
| 49 | Shanxi2019-13 | Female | 28 |  | √ |  |  |  |  |  |  |  |  |  |  |  | 2019.04.24 | HAdV-8 [P8H8F8] |  |
| 50 | Shanxi2019-14 | Female | 56 | √ |  |  |  |  |  |  |  |  |  | √ |  |  | 2019.04.24 | HAdV-4 [P4H4F4] |  |
| 51 | Shanxi2019-15 | Male | 34 | √ |  |  |  |  | √ |  |  | √ |  | √ |  |  | 2019.04.24 | HAdV-3 [P3H3F3] |  |
| 52 | Shanxi2019-18 | Male | 76 | √ | √ | √ |  | √ |  |  |  |  |  |  |  |  | 2019.04.24 | HAdV-8 [P8H8F8] |  |
| 53 | Shanxi2019-20 | Male | 20 | √ | √ |  |  | √ |  |  |  |  |  |  |  |  | 2019.04.29 | HAdV-8 [P8H8F8] |  |
| 54 | Shanxi2019-21 | Female | 37 | √ | √ | √ |  | √ |  | √ |  |  |  |  |  |  | 2019.04.30 | HAdV-8 [P8H8F8] |  |
| 55 | Shanxi2019-23 | Male | 30 | √ | √ |  |  | √ |  |  |  | √ |  | √ |  |  | 2019.05.07 | HAdV-8 [P8H8F8] |  |
| 56 | Shanxi2019-24 | Female | 60 | √ | √ |  |  | √ |  |  |  | √ |  | √ |  |  | 2019.05.08 | HAdV-8 [P8H8F8] |  |
| 57 | Shanxi2019-25 | Male | 28 | √ | √ |  |  |  |  |  |  | √ |  |  |  |  | 2019.05.08 | HAdV-8 [P8H8F8] |  |
| 58 | Shanxi2019-26 | Male | 30 | √ | √ |  |  |  |  |  |  | √ |  | √ |  |  | 2019.05.14 | HAdV-37 [P37H37F37] |  |
| 59 | Shanxi2019-28 | Female | 30 |  |  |  |  |  | √ |  |  |  |  |  |  |  | 2019.09.09 | HAdV-8 [P8H8F8] |  |
| 60 | Shanxi2019-30 | Male | 50 | √ | √ |  |  |  |  |  |  |  |  | √ |  |  | 2019.09.09 | HAdV-8 [P8H8F8] |  |
| 61 | Shanxi2019-31 | Female | 26 | √ | √ |  |  |  |  |  |  | √ |  | √ |  |  | 2019.09.09 | HAdV-8 [P8H8F8] |  |
| 62 | Shanxi2019-32 | Male | 46 | √ | √ |  |  |  | √ |  |  |  |  |  | √ |  | 2019.09.09 | HAdV-8 [P8H8F8] |  |
| 63 | Shanxi2019-34 | Female | 59 | √ |  |  |  |  |  |  |  | √ |  |  |  |  | 2019.09.16 | HAdV-8 [P8H8F8] |  |

Supplementary Table 3. The global representative strains of HAdV used in this study

| Genotype | Country | Penton base | |  | Hexon | |  | Fiber | |
| --- | --- | --- | --- | --- | --- | --- | --- | --- | --- |
|  |  | Year | No. of seq |  | Year | No. of seq |  | Year | No. of seq |
| HAdV-3 | CHN | 2004-2020 | 24 |  | 2004-2020 | 30 |  | 2004-2020 | 28 |
|  | JPN | 1988-2004 | 3 |  | 1988-2004 | 3 |  | 1988-2004 | 3 |
|  | KOR | 2015 | 1 |  | 1999,2015 | 2 |  | 1998-2015 | 6 |
|  | USA | 1953-2008 | 11 |  | 1953-2008 | 11 |  | 1953-2008 | 11 |
| HAdV-4 | CHN | 2008-2014 | 3 |  | 2008-2014 | 5 |  | 2008-2014 | 3 |
|  | EGY | 1968 | 1 |  | 1968 | 1 |  | 1968 | 1 |
|  | FRA | 1978 | 1 |  | 1978 | 1 |  | 1978 | 1 |
|  | JPN | 1981-2001 | 4 |  | 1981-2001 | 4 |  | 1981-2001 | 4 |
|  | SGP | 2016 | 1 |  | 2016 | 1 |  | 2016 | 1 |
|  | SWE | 2000 | 1 |  | 2000 | 1 |  | 2000 | 1 |
|  | USA | 1953-2019 | 29 |  | 1953-2019 | 26 |  | 1953-2019 | 29 |
| HAdV-8 | CHN | 2016,2018 | 2 |  | 2016,2018 | 2 |  | 2016,2018 | 2 |
|  | DEU | 2003-2013 | 5 |  | 2003-2013 | 5 |  | 2003-2013 | 5 |
|  | IRN | 2018 | 1 |  | 2018 | 1 |  | 2018 | 1 |
|  | JPN | 1991-2014 | 7 |  | 1991-2014 | 8 |  | 1991-2014 | 7 |
|  | SAU | 2011 | 1 |  | 2011 | 1 |  | 2011 | 1 |
|  | SGP | 2011 | 1 |  | 2011 | 1 |  | 2011 | 1 |
|  | SWE | 1986-1990 | 3 |  | 1986-1990 | 3 |  | 1986-1990 | 3 |
|  | USA | 1955-2012 | 4 |  | 1955-2012 | 4 |  | 1955-2012 | 4 |
|  | VNM | 2017 | 1 |  | 2017 | 1 |  | 2017 | 1 |
|  | TUN | / | / |  | / | / |  | 2000-2013 | 3 |
| HAdV-37 | JPN | 1991-2020 | 9 |  | 1991-2020 | 15 |  | 1991-2020 | 13 |
|  | NED | 1976 | 1 |  | 1976 | 1 |  | 1976 | 1 |
|  | TUN | / | / |  | / | / |  | 2013 | 1 |
| HAdV-53 | DEU | 2005 | 1 |  | 2005 | 1 |  | 2005 | 1 |
|  | JPN | 1989-2004 | 5 |  | 1989-2004 | 5 |  | 1989-2004 | 5 |
|  | USA | 2017-2018 | 2 |  | 2017-2018 | 2 |  | 2017-2018 | 2 |
| HAdV-64 | JPN | 1992,1999 | 2 |  | 1992,1999 | 2 |  | 1992-2002 | 3 |
|  | USA | 1955-1993 | 4 |  | 1955-1993 | 4 |  | 1955-1993 | 4 |
| HAdV-85 | JPN | 2015,2017 | 2 |  | 2015,2017 | 2 |  | 2015,2017 | 2 |

Supplementary Table 4. Genome coding annotation of two HAdV-85 strains in this study

| Gene | Product | Coding annotation |
| --- | --- | --- |
|  |  | Shanxi2016-39 / Shanxi2016-70 |
| E1A | Early E1A 13S | 570-1139,1171-1425 |
| E1B | 19kDa protein | 1578-2126 |
|  | 55kDa protein | 1883-3370 |
| IX | pIX | 3455-3859 |
| IVa2 | pIVa2 | (3903-4949) c |
| E2B | DNA polymerase | (5006-8526,13492-13504) c |
|  | pTP | (8326-10223,13492-13504) c |
| L1 | 52/55K protein | 10639-11763 |
|  | pIIIa | 11786-13477 |
| L2 | penton base | 13531-15090 |
|  | pVII | 15094-15684 |
|  | pV | 15717-16721 |
|  | pX | 16751-16975 |
| L3 | pVI | 17031-17735 |
|  | hexon | 17776-20661 |
|  | protease | 20664-21287 |
| E2A | DNA binding protein | (21342-22814) c |
| L4 | 100kDa protein | 22831-25029 |
|  | 22kDa protein | 24812-25225 |
|  | pVIII | 25530-26213 |
| E3 | 12.2kDa protein | 26214-26534 |
|  | CR1-alpha | 26488-27081 |
|  | gp19K | 27078-27551 |
|  | CR1-beta | 27520-28827 |
|  | CR1-gamma | 28854-29684 |
|  | RID-alpha | 29692-29967 |
|  | RID-beta | 29970-30359 |
|  | 14.7kDa protein | 30379-30744 |
| L5 | fiber | 30998-32086 |
| E4 | 34kDa protein | (32362-33240) c |
|  | Orf4 | (33170-33532) c |
|  | Orf3 | (33535-33888) c |
|  | Orf2 | (33885-34277) c |
|  | Orf1 | (34318-34695) c |
